# Supplementary material for: Prostate Cancer Morphologies: Cribriform Pattern and Intraductal Carcinoma Relations to Adverse Pathological and Clinical Outcomes—Systematic Review and Meta-Analysis
Source: Cancers (Basel). 2023 Feb 21;15(5):1372. doi: 10.3390/cancers15051372 (PMC10000112; doi:10.3390/cancers15051372)
Supplement: Supplementary file 1 [file cancers-15-01372-s001.zip › cancers-2212116-supplementary.pdf]

cancers

MDPI

Systematic Review

Prostate Cancer Morphologies: Cribriform Pattern and Intraductal Carcinoma Relations to Adverse Pathological and Clinical Outcomes—Systematic Review and Meta-Analysis

Rafał Osiecki, Mieszko Kozikowski, Beata Sarecka-Hujar, Michał Pyzlak and Jakub Dobruch

Table S1. Characteristics of included studies - after correction

Author	Sample collection period	Study desing	CR/IDC	ISUP grading guildnes	IDC definition	Revision of pathological slides	IHC	Sample size (N)	Cohort GG	number CP/IDC	Outcome measures reported	Monitoring measures	Outcome measured additionally extracted for meta-analysis	BCR definition	Follow-up duration	LND met, MET, Recurrence - diagnosis	Exclusion criteria	Other infotmation	Comments
Sarbay et al. [8]	2006-2012	retrospective case control study-single centre	CP	2005 ISUP	NA	NOT STATED	YES	185 (consecutive)	GG 1-98, GG 2 + 3-78, GG 5-9	115	EPE, SM	NA	NA	NA	NA	NA	NOT STATED	NA	Authors enrolled ISUP 1 patients following 2005 ISUP recommendations
Dong et al. [16]	1993-1997	retrospective case control study--single centre	CP	2005 ISUP	NA	YES- 2 reviewers+select slides with the highest Gleason grade were reevaluated by 2 additional reviewers	NOT STATED	241 cases with ISUP 2-4 from an original consecutive sample of 755 cases	GG 2 - 183, GG 3 - 40, GG 4-18	165	BCR, MET	BCR, MET	NA	<1999 ≥ 0,5ng/ml, 1999-2006 ≥0,2ng/ml, ≥2007 ≥0,1ng/ml	Median: BCR -5,5 years; MET-10,0 years	Prostate cancer recurrence in a lymph node or at a distant site by clinical interpretation or radiological evidence	Neoadjuvant therapy, adjuvant therapy before biochemical failure, unavailable histologic slides, no postoperative follow-up, and postoperative PSA persistence, GG5.	GS, architectural pattern, PSA, age, pT, SM, and prostate weight were included in multivariante analysis as potential confunders	EPE and SVI used as a prognostic indicator in multivariante analysis
Choy et al. [12]	2003-2006	retrospective case control study-single centre	CP	2014 ISUP	NOT STATED	YES- 2 reviewers	NO	350 cases from a original cohort of 585 consecutive patients	GG 2-287, GG 3-63	154	5 year BCR - free survival	5 year BCR-free survival	NA	Any postoperative PSA level of ≥0.2 ng/mL	Median 74 months (IQR 42-85) with death as an end point	NA	Prior hormonal or radiation therapy	Age, PSA, SM, pT, percent GP 4, and architectural types were included in multivariante analysis as potential confunders	Only ISUP 2 and 3 patients were analysed in terms of BCR.

Greenland et al. [11]	July 2015-July 2018	retrospective cohort study-single centre	expansile CP	NOT STATED	NOT STATED	NO- only pathology reports were reviewed	NO	110	GG 2-77, GG 3-33	55	IDC, EPE, SVI, LNs met, BCR	BCR free survival	NA	2 consecutive PSA measurements ≥ 0.2 ng/mL starting 8 weeks after surgery.	Median: CP 360,5 days; glomerulation pattern 407 days	NA	Cases with pattern 5 or with both the expansile cribriform and glomerulations patterns.	Authors provided additional analysis of DECIPHER score in patients positive for CP and glomerulation pattern - see disscussion	Presented data didn't allow for the inclusion of both CP and IDC into statistical analysis.
Luo et al. [7]	January 2015-January 2016	retrospective case control study- -single centre	ICLs	2014 ISUP	NA	YES - to identify ICLs	YES	85 (consecutive)	GG 1-15, GG 2-51, GG 3-13, GG 4-1, GG 5-5,	40	BCR, LR, MET/DSD	BCR, LR, MET/DSD	EPE, SVI, LNs met	PSA concentration greater than 0.1 ng/mL on 2 consecutive occasions	Median: 20 months(range 2–38)	NOT STATED	A history of neoadjuvant/adjuvant therapies	Age, ethnicity, PSA , and tumor load were included in multivariante analysis as potential confunders	The study also evaluatated local recurrence (see discussion). No diagnostic criteria for LC were presented. Authors additionally performed meta-analysis on the association of LC with adverse automes.
Kir et al. [9]	2006-2013	retrospective case control study-single centre	CP	2005 ISUP	NA	YES- 2 reviewers	YES	233 (consecutive)	GG 1-109, GG 2 -85, GG 3-26, GG 5(4+5)-9, GG 5(5+4)-4	144	BCR	BCR	NA	PSA level above 0.1 ng/mL	Median: 3.5 years (range 1–7)	NA	A history of a cryotherapy, radiotherapy, or androgen deprivation	GS, pT, SM , CP, PSA were included in multivariante analysis as potential confunders	Cohort from this study overlaped with Sarbay et al. Those studies differ in end-points.
Kweldam et al. [13]	1985-2013	retrospective nested paired case control study--	CP	1985-2005 = Classic Gleason	NA	YES- 2 reviewers	YES	161 (52 cases and 109 control	GG 2 + 3- 161	83	BCR-free survival,	BCR-free survival, MET,	NA	PSA level of 0.2 ng/ml, assessed at two	Median: BCR CP (+) 34 months(IQR 11–88); CP(-) 120	Presence of prostate cancer in a lymph node or at a distant site,	NOT STATED	Cases and controls were matched for age at time of surgery, PSA.	LNs met were used as a covariate in multivariate analysis of MET

		single centre		Grading, >2005=ISUP2005				s from a entire cohort of 535 GS 7 patients)			MET, DSD, OS	DSD, OS		consecutive time points > 3 months apart after radical prostatectomy.	months(IQR 140–170). Met/DSD CP(+) 88 months (IQR 42-160;, CP(-) 120 months (IQR 120-180)	with radiologic or pathologic confirmation		Age, PSA level, GS, pT stage, SM, LNs status, Gleason grade 4 patterns, intraductal carcinoma, and tertiary Gleason grade 5 were included in multivariable analysis as potential confunders	and DSD. BCR-free survival wasn't included as result of incomplete data.
Iczkowski et al. [10]	NOT STATED	retrospective paired case-control-multicentre	CP	2005 ISUP	NA	YES-Consensus conferences held by 4 reviewers	NO	153 (76 cases, 77 controls)	NA	58	PSA failure (BCR)	PSA failure (BCR)	NA	PSA rise to 0.2 ng/mL (0.2 µg/L) or more, without evidence of later, lower measurements that would invalidate the 0.2 level	Median: PSA failure 2,156 days(range 35-4,612); non PSA failure 2,017 days (range 398-4,198)	NA	Cryotherapy, radiotherapy, or androgen deprivation before failure	Cases and controls were matched for follow up duration (primary criterion), age, grade, pT, SM (secondary criteria).	NA
Kryvenko et al. [5]	1993-2010`	retrospective paired case control study-multicentre	IDC	2005 ISUP	Guo and Epstein	YES- 2 reviewers	NOT STATED	302 (140 cases, 162 controls)	GG 2 + 3-302	116	LNs met	NA	NA	NA	NA	NA	Prior hormonal or radiation therapy	The case-control pairs were matched for were matched for the tumor extension-cancers limited to the prostate (pT2) and with extension beyond the prostate (pT3).	NA

Trinh et al. [3]	1993-2011	retrospective case control study-multicentre	IDC	ISUP2014	Guo and Epstein	YES- 2 reviewers	YES	85	GG 1-7, GG 2-16, GG 3-37, GG 4-6, GG 5-19	65	site of initial CR, time to CR, MET, CSS,	MET, CSS	EPE, SVI, LNs met	NA	Mean: IDC (+)-109.5 months (55.9-SD); IDC (-)-120.8 months (49.4- SD)	LR - peri-prostatic involvement after surgery as confirmed by imaging, biopsy, and/or suspect results fromDRE, followed by exclusive radiotherapy to the prostatic lodge leading to a serum PSA level reduction without concurrent hormone therapy.Other types of CR had to be confirmed by radiology and/or biopsy.	Chemotherapy pior to CR	Recorded clinical data included age at diagnosis, date of diagnosis, PSA, recurrence dates and site, and follow-up time until mortality or last contact. If relevant, cause of death was recorded	Author additionally investigated the impact of pre- CR treatment on CSS.
Dinerman et al. [2]	2004-2013	retrospective population based-study	IDC	NA	NOT STATED	NA	NOT STATED	159,777	≤GG 1-59,553, ≥GG 2 -100,204	242	DSD, OM	DSD, OM	NA	NA	Median; IDC(+) 3,9 years (IQR 2,2-6,6); IDC(-) 4,8 years(IQR 2,6-7,0)	NA	Prostatic ductal carcinoma	pT stage, GS, IDC, LNs status were included in multivariable analysis as potential confunders	As a result of different study desing, the study excluded from quantitative analysis
Kato et al. [4]	2005 - 2013	retrospective case-control study-multicentre	IDC	2014 ISUP	McNeal and Yemoto	1 reviewer	NOT STATED	1019 (consecutive)	GG 1-163, GG 2-499, GG 3-220, GG 4-	157	BCR free survival	BCR free survival	NA	Continuously rising PSA level >0.2 ng/mL.	Median: 82 months (range 0.7-148)	NA	Missing data or slides	Grade group, age, PSA level, pT stage, the presence of Gleason 5 pattern, IDC, surgical margin status were	As a result of lack of detailed statistical data, the study was excluded from quantitative analysis.

									40, GG 5-97									included in multivariable analysis as potential confunders	
Miyai et al. [1]	2006-2012	retrospective case-control study-single centre	IDC	NOT STATED	(1) solid or dense cribriform (less than 50% lumen in a duct) intraductal lesions or (2) loose cribriform (50% and more than 50% lumen in a duct) or micropapillary intraductal lesions with prominent nuclear pleomorphism	NO- only pathology reports were reviewed	YES	901 (consecutive)	GG ≤3 - 753, GG ≥4- 148	155	EPE, SVI, BCR	BCR	LNs met	Serum PSA ≥ 0.2 ng/ml after a previously undetectable serum PSA value.	Median 17 months (range 1-86)	NA	Adjuvant or neoadjuvant hormone therapy or adjuvant radiotherapy.	Preoperative PSA level, GS PSM, EPE, SVI, LVI, LNs met, pT stage, IPL were included in multivariable analysis as potential confunders	BCR information in 827 cases

					(nuclear size greater than 6× normal) and/or non-focal comedo necrosis.														
Flood et al. [15]	2010-2016	retrospective case control study-single centre	CP/ICD	2014 ISUP	Guo and Epstein	YES- 2 reviewers	YES	28	GG 5-28	27	BCR	BCR	NA	Postoperative PSA value of 0.2 ng/mL or higher	Mean time to BCR 107 days (IQR; 70-400 days)	NA	Adjuvant post-operative radiation or androgen deprivation before BCR	Age, PSA, PSAD, EPE, SVI, LVI and different Pca patterns were included in multivariable analysis as potential confunders	NA
Hollemans et al. [14]	2010-2017	retrospective case control study-single centre	CP/IDC	2016 WHO	IDC-identified if cribriform structures were clearly continuous with pre-existing glands lined by normal basal epithelium, or	YES- 2 reviewers	YES	420	GG 2-420	228	BCR-free survival, MET/DSD	BCR-free survival	EPE, SVI, LNs met	A PSA level of ≥0.2 ng/ml measured at two separate points in time at least 3 months apart when PSA had been undetectable after surgery, or as a PSA increase of >2.0 ng/ml whenever	Median: 59.6 months (IQR 17.5–113.9)	LNS met and MET were confirmed by biopsy or multidisciplinary consensus	Hormonal, radiation or viral therapy prior to surgery	Age, PSA, pT, percentage Gleason 4, tertiary Gleason pattern 5, SM, LNs met, GG were included in multivariable analysis as potential confunders	Meta- analysis included only ISUP 2 patients

					containing corpora amylacea									serum PSA had not declined to zero after surgery.					
Trudel et al. [6]	1998-2001	case control retrospective study-single centre	LC/ IDC	2005 ISUP	Guo and Epstein	YES- 2 reviewers	YES	246	GG 1-127, GG 2+3-101, GG 4+5-18	80	BCR -free rate	BCR-free rate	NA	Rising PSA > 0.2 ng/ml at 2 occasions after prostatectomy.	Median 130.6 months (range 1.5–175.3)	NA	Incomplete follow up	Prostate weight, GS, SM, pT were included in multivariable analysis as potential confunders	As a result of lack of detailed statistical data, the study was excluded from quantitative analysis.

CP=cribriform pattern, IDC= intraductal carcinoma, ICLs- invasive cribriform lesions, LC- large cribriform ISUP= International Society of Urological Pathology, IHC= immunohistochemical staining, GS=Gleason score, GG =Gleason grade, EPE= extraprostatic extension, SVI-=seminal vesicle invasion, SM=surgical margin, LNs met= lymph node metastasis, BCR= biochemical recurrence, MET= distant metastasis, DSD= disease specific death, OS= overall survival, LR= local recurrence, DRE= digital.

Table S2. ROB case-control studies

NOS- case control studies	Luo et al. [7]	Dong et al. [16]	Sarabay et al. [8]	Miyai et al. [1]	Iczkowski et al. [10]	Flood et al. [15]	Kweldam et al. [13]	Choy et al. [12]	Kir et al. [9]	Hollemans et al. [14]	Kryvenko et.al. [5]	Trinh et al. [3]
Is the case definition adequate?	1	1	1	1	1	1	1	1	1	1	1	1
Representativeness of the cases	1	1	0	1	0	1	1	1	1	1	1	1
Selection of Controls	1	1	1	1	1	1	1	1	1	1	1	1
Definition of Controls	1	1	1	1	1	1	1	1	1	1	1	1
Comparability of cases and controls on the basis of the design or analysis	2	2	1	2	2	2	2	2	2	2	2	2
Ascertainment of exposure	1	1	1	1	1	1	1	1	1	1	1	1
Same method of ascertainment for cases and controls	1	1	1	1	1	1	1	1	1	1	1	1
TOTAL	8	8	6	9	7	8	8	8	8	8	8	8

NOS=The Newcastle-Ottawa Scale

Table S3. ROB cohort study

NOS - cohort study	Greenland et al. [11]
Representativeness of the exposed cohort	1
Selection of the non-exposed cohort	1
Ascertainment of exposure	1
Demonstration that outcome of interest was not present at start of study	0
Comparability of cohorts on the basis of the design or analysis	1
Assessment of outcome	1
Was follow-up long enough for outcomes to occur?	0
Adequacy of follow up of cohorts	0
TOTAL	5

NOS= The Newcastle-Ottawa Scale

Table S4. The results of Egger's and Begg's tests.

	Egger's test			Begg's test	
	**Intercept**	**95% CI**	***p***	**Kendall's tau**	***p***

EPE	0.076	-7.408 to 7.561	0.981	0.428	0.179
SVI	-0.543	-5.667 to 4.581	0.796	0.143	0.773
LNs met	0.913	-0.670 to 2.497	0.198	0.238	0.562
BCR	1.797	-3.152 to 6.746	0.419	0.278	0.358
MET/DSD	1.726	-2.912 to 6.365	0.321	0.400	0.483

EPE= extraprostatic extension, SVI= seminal vesicle invasion, LNs met= lymph node metastasis, BCR= biochemical recurrence, MET=distant metastasis, DSD= disease-specific death, CI= confidence interval

References

1. Miyai, K.; Divatia, M.K.; Shen, S.S.; Miles, B.J.; Ayala, A.G.; Ro, J.Y. Clinicopathological analysis of intraductal proliferative lesions of prostate: Intraductal carcinoma of prostate, high-grade prostatic intraepithelial neoplasia, and atypical cribriform lesion. *Hum. Pathol*. **2014**, *45*, 1572–1581. https://doi.org/10.1016/j.humpath.2014.03.011.
2. Dinerman, B.F.; Khani, F.; Golan, R.; Bernstein, A.N.; Cosiano, M.F.; Margolis, D.J.; Hu, J.C. Population-based study of the incidence and survival for intraductal carcinoma of the prostate. *Urol. Oncol*. **2017**, *35*, 673.e9–673.e14. https://doi.org/10.1016/j.urolonc.2017.08.015.
3. Trinh, V.Q.; Sirois, J.; Benzerdjeb, N.; Mansoori, B.K.; Grosset, A.A.; Albadine, R.; Latour, M.; Mes-Masson, A.M.; Hovington, H.; Bergeron, A.; et al. The impact of intraductal carcinoma of the prostate on the site and timing of recurrence and cancer-specific survival. *Prostate* **2018**, *78*, 697–706. https://doi.org/10.1002/pros.23513.
4. Kato, M.; Hirakawa, A.; Kobayashi, Y.M.; Yamamoto, A.; Ishida, R.; Sano, T.; Kimura, T.; Majima, T.; Ishida, S.; Funahashi, Y.; et al. The influence of the presence of intraductal carcinoma of the prostate on the grade group system's prognostic performance. *Prostate* **2019**, *79*, 1065–1070. https://doi.org/10.1002/pros.23818.
5. Kryvenko, O.N.; Gupta, N.S.; Virani, N.; Schultz, D.; Gomez, J.; Amin, A.; Lane, Z.; Epstein, J.I. Gleason score 7 adenocarcinoma of the prostate with lymph node metastases: Analysis of 184 radical prostatectomy specimens. *Arch. Pathol. Lab. Med*. **2013**, *137*, 610–617. https://doi.org/10.5858/arpa.2012-0128-OA.
6. Trudel, D.; Downes, M.R.; Sykes, J.; Kron, K.J.; Trachtenberg, J.; van der Kwast, T.H. Prognostic impact of intraductal carcinoma and large cribriform carcinoma architecture after prostatectomy in a contemporary cohort. *Eur. J. Cancer* **2014**, *50*, 1610–1616. https://doi.org/10.1016/j.ejca.2014.03.009.
7. Luo, X.; Khurana, J.S.; Jhala, N.; Zhao, H.; Wang, H. The Association of Invasive Cribriform Lesions With Adverse Prostatic Adenocarcinoma Outcomes: An Institutional Experience, Systematic Review, and Meta-analysis. *Arch. Pathol. Lab. Med*. **2019**, *143*, 1012–1021. https://doi.org/10.5858/arpa.2017-0582-RA
8. Sarbay, B.C.; Kir, G.; Topal, C.S.; Gumus, E. Significance of the cribriform pattern in prostatic adenocarcinomas. *Pathol. Res. Pract*. **2014**, *210*, 554–557. https://doi.org/10.1016/j.prp.2014.03.003.
9. Kir, G.; Sarbay, B.C.; Gümüş, E.; Topal, C.S. The association of the cribriform pattern with outcome for prostatic adenocarcinomas. *Pathol. Res. Pract*. **2014**, *210*, 640–644. https://doi.org/10.1016/j.prp.2014.06.002.
10. Iczkowski, K.A.; Torkko, K.C.; Kotnis, G.R.; Wilson, R.S.; Huang, W.; Wheeler, T.M.; Abeyta, A.M.; La Rosa, F.G.; Cook, S.; Werahera, P.N.; et al. Digital quantification of five high-grade prostate cancer patterns, including the cribriform pattern, and their association with adverse outcome. *Am. J. Clin. Pathol*. **2011**, *136*, 98–107. https://doi.org/10.1309/AJCPZ7WBU9YXSJPE
11. Greenland, N.Y.; Cowan, J.E.; Zhang, L.; Carroll, P.R.; Chan, E.; Stohr, B.A.; Simko, J.P. Expansile cribriform Gleason pattern 4 has histopathologic and molecular features of aggressiveness and greater risk of biochemical failure compared to glomerulation Gleason pattern 4. *Prostate* **2020**, *80*, 653–659. https://doi.org/10.1002/pros.23977.
12. Choy, B.; Pearce, S.M.; Anderson, B.B.; Shalhav, A.L.; Zagaja, G.; Eggener, S.E.; Paner, G.P. Prognostic Significance of Percentage and Architectural Types of Contemporary Gleason Pattern 4 Prostate Cancer in Radical Prostatectomy. *Am. J. Surg. Pathol*. **2016**, *40*, 1400–1406. https://doi.org/10.1097/PAS.0000000000000691.
13. Kweldam, C.F.; Wildhagen, M.F.; Steyerberg, E.W.; Bangma, C.H.; van der Kwast, T.H.; van Leenders, G.J. Cribriform growth is highly predictive for postoperative metastasis and disease-specific death in Gleason score 7 prostate cancer. *Mod. Pathol*. **2015**, *28*, 457–464. https://doi.org/10.1038/modpathol.2014.116.
14. Hollemans, E.; Verhoef, E.I.; Bangma, C.H.; Rietbergen, J.; Roobol, M.J.; Helleman, J.; van Leenders, G.J.L.H. Clinical outcome comparison of Grade Group 1 and Grade Group 2 prostate cancer with and without cribriform architecture at the time of radical prostatectomy. *Histopathology* **2020**, *76*, 755–762. https://doi.org/10.1111/his.14064.
15. Flood, T.A.; Schieda, N.; Sim, J.; Breau, R.H.; Morash, C.; Belanger, E.C.; Robertson, S.J. Evaluation of tumor morphologies and association with biochemical recurrence after radical prostatectomy in grade group 5 prostate cancer. *Virchows Arch*. **2018**, *472*, 205–212. https://doi.org/10.1007/s00428-017-2241-9.
16. Dong, F.; Yang, P.; Wang, C.; Wu, S.; Xiao, Y.; McDougal, W.S.; Young, R.H.; Wu, C.L. Architectural heterogeneity and cribriform pattern predict adverse clinical outcome for Gleason grade 4 prostatic adenocarcinoma. *Am. J. Surg. Pathol*. **2013**, *37*, 1855–1861. https://doi.org/10.1097/PAS.0b013e3182a02169
